# Nonfatal Hyperammonemic Encephalopathy as a Late Complication of Roux-en-Y Gastric Bypass

**DOI:** 10.1155/2019/9031087

**Published:** 2019-07-01

**Authors:** Juan D. Salcedo, Jordan S. Goldstein, Jose M. Quinonez, Maria Antonietta Mosetti

**Affiliations:** ^1^Department of Internal Medicine, University of Miami Miller School of Medicine, Miami, FL, USA; ^2^University of Miami Miller School of Medicine, Miami, FL, USA; ^3^Division of Hospital Medicine, University of Miami Medical Towers, University of Miami Miller School of Medicine, Miami, FL, USA

## Abstract

Roux-en-Y gastric bypass (RYGB) is the most common weight loss procedure performed in the US. Gastric bypass–related hyperammonemia (GaBHA) is a potentially fatal entity, characterized by encephalopathy associated with hyperammonemia and various nutritional deficiencies, which can present at variable time intervals after RYGB. Twenty-five cases of hyperammonemic encephalopathy after bariatric surgery have been previously reported in the literature. We describe the case of a 48-year-old Hispanic woman with no prior history of liver disease, presenting with nonfatal hyperammonemic encephalopathy as a late postoperative complication 20 years after undergoing a RYGB. Hyperammonemic encephalopathy in the absence of known hepatic dysfunction presents a diagnostic dilemma. An early diagnosis and intervention are crucial to decrease morbidity and mortality.

## 1. Introduction

Roux-en-Y gastric bypass (RYGB) is the most common weight loss procedure performed in the US [1, 2]. It achieves successful and sustained weight loss in morbidly obese patients, through restrictive and malabsorptive mechanisms [3].

Although RYGB has lower morbidity and mortality rates compared to other bariatric procedures, there are numerous postoperative complications which may occur [4]. Early postoperative complications include gastrointestinal leaks, venous thromboembolic disease, pulmonary and cardiovascular complications, and tracheal reintubation [5]. The average 30-day mortality rate reported for RYGB is 0.2% [6]. Late postoperative complications include anastomotic stricture, bowel obstruction, fistula formation, intussusception, marginal ulcers, gallstone formation, dumping syndrome, hypoglycemia, malnutrition, fat malabsorption, and vitamin deficiencies [7].

Neurologic complications after bariatric surgery have a reported prevalence of 5% [8], including optic neuropathy, polyradiculoneuropathy, myelopathy, and encephalopathy. Of these, encephalopathy is a rare complication, usually occurring early, and generally attributed to thiamine deficiency [9]. Gastric bypass–related hyperammonemia (GaBHA) has been recently recognized as a life-threatening distinct entity, characterized by encephalopathy associated with hyperammonemia, elevated plasma glutamine, hypoalbuminemia, reactive hypoglycemia, low zinc, and other nutritional deficiencies. This condition has a high fatality rate reported around 50% and has a predilection for middle-aged women without prior liver disease who have achieved successful weight loss after RYGB [10]. It can occur at variable time intervals after RYGB, ranging from 1 month to 28 years after the surgery [11]. Hyperammonemic encephalopathy in the absence of known hepatic dysfunction presents a diagnostic dilemma. Therefore, an early diagnosis and intervention are crucial to prevent a rapid decline that may occur when the diagnosis is unrecognized.

## 2. Methods

We conducted a MEDLINE database query by combining the search terms “Hyperammonemia”, “Encephalopathy”, and “Bariatric surgery”. The resultant seven articles [10–16] were pulled from the literature. English language article titles and abstracts were screened and the appropriate articles reviewed. As far as we could determine in our search, twenty-five cases of hyperammonemic encephalopathy after bariatric surgery have been previously reported in the literature.

## 3. Case Presentation

A 48-year-old Hispanic female with a past medical history of multiple bariatric surgical procedures, presented with increasing lower extremity edema secondary to worsening chronic lymphedema.

During her hospitalization she developed altered mental status in the setting of elevated serum ammonia levels, despite having no history of liver disease.

Vertical banded gastroplasty was initially performed at age 28, but it failed to produce adequate weight loss. Consequently, three years later, she underwent open surgical revision which was converted to an open RYGB with concomitant cholecystectomy. She had lost over 100 kg since the first bariatric procedure (166 kg preoperatively to 63 kg on presentation). However, she developed multiple complications, including chronic lower extremity lymphedema, small bowel obstruction requiring multiple surgical interventions, malabsorption secondary to short bowel syndrome, multiple vitamin deficiencies, wet beriberi requiring chronic thiamine replacement, osteoporosis causing a left hip fracture, and chronic low back pain.

In July 2018, she initially presented to the emergency department with increasing lower extremity edema secondary to chronic lymphedema. On arrival, her vital signs were within normal limits. Her physical exam was remarkable for bilateral lower extremity pitting edema extending to the hips and an otherwise unremarkable exam. A chest x-ray disclosed no cardiomegaly, pulmonary congestion or pleural effusion. Her last echocardiogram showed an ejection fraction of 60% with no wall motion abnormalities.

During the admission, the patient received intravenous bumetanide with significant improvement. However, she developed waxing and waning episodes of confusion, and on day 3 of her hospitalization became overtly encephalopathic. She was disoriented to person, time, and space, with bizarre affect as well as incoherent speech, unable to recognize her close relatives. On physical exam patient was lethargic and had asterixis. According to her family members, the patient had previous episodes where she would become lethargic, but never to this degree.

Laboratory results were significant for an elevated plasma ammonia level of 173 *μ*mol/L (normal range 11 - 50 *μ*mol/L), despite downtrending transaminases (aspartate transaminase of 32 U/L and alanine transaminase of 55 U/L). Results were also significant for macrocytic anemia (hemoglobin 10.3 g/dL and mean corpuscular volume 107/5), a low serum albumin of 2.3 g/dL, low serum protein of 4.1 g/dL, alkaline phosphatase of 122 U/L, total bilirubin of 0.5 mg/dL, and an elevated international normalized ratio (INR) of 1.65. Serum glucose and zinc levels were borderline low at 62 and 54 (normal range 56 – 134 *μ*g/dL), respectively. The laboratory findings are further outlined in Table 1. Additionally, her plasma salicylate level, acetaminophen level, and urine toxicology were all negative. Despite a history of vitamin deficiencies, she was receiving chronic thiamine and vitamin B12 replacement at home, with normal values on admission. Noncontrast computed tomography (CT) imaging of the head disclosed no evidence of acute infarction, intracranial hemorrhage, or mass effect. The patient had no history of prior liver conditions or cirrhosis, and even though she presented elevation of transaminases and the INR, these abnormalities resolved once her condition improved. The patient had no other laboratory findings for compatible signs of cirrhosis (no thrombocytopenia or hyperbilirubinemia), neither physical stigmata suggestive of acute or chronic liver disease. Abdominal ultrasound was performed to further elucidate liver panel abnormalities and demonstrated only mild hepatic steatosis with no evidence of cirrhosis or ascites.

She was treated with supportive care and lactulose. The following day she had numerous bowel movements, and her serum ammonia level came down to 24 *μ*mol/L. Given the fast clinical improvement and resolution of laboratory abnormalities after supportive treatment, a liver biopsy was not indicated. Once her clinical condition improved and her mental status returned to normal, the patient was discharged from the hospital.

## 4. Discussion

Ammonia is extremely toxic to the central nervous system [17]. It is a bi-product of protein catabolism from amino acid deamination (whether from a high-protein diet, prolonged starvation, or naturally produced by gut flora) [18]. The urea cycle catabolizes toxic ammonia into urea, a process that occurs predominantly in the liver. Urea is nontoxic and is ultimately excreted by the kidneys [18].

**Figure 1:**
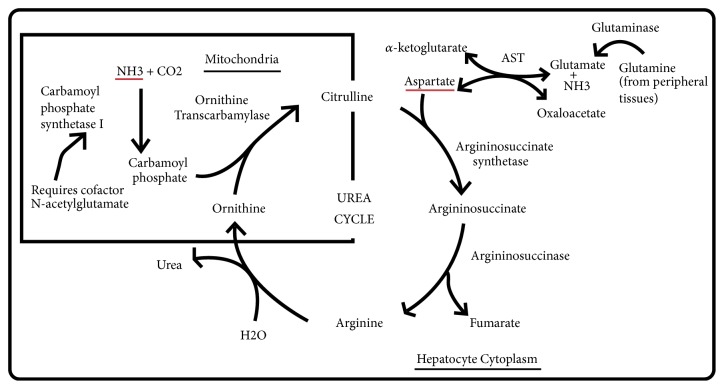
Urea cycle. Ammonia is the source of the first amino group in the urea cycle, required in the synthesis of carbamoyl phosphate (CPS), which happens in the hepatocyte mitochondria. The second amino group comes from glutamate in the formation of aspartate, which happens in the hepatocyte cytoplasm. This reaction produces *α*-ketoglutarate, which then becomes available for the Krebs cycle. The synthesis of argininosuccinate links the Krebs cycle and urea cycle. In the peripheral tissues, glutamate accepts free ammonia from amino acid catabolism. Glutamine transports ammonia from the peripheral tissues to the liver, where glutamine is broken back to glutamate and ammonia via glutaminase. AST, aspartate transaminase.

In the urea cycle (Figure 1), ammonia is the source of the first amino group, required in the synthesis of carbamoyl phosphate, which happens in the hepatocyte mitochondria. The second amino group comes from glutamate in the formation of aspartate, which happens in the hepatocyte cytoplasm. This reaction produces *α*-ketoglutarate, which then becomes available for the Krebs cycle [18]. In the muscle and peripheral tissues (including the brain astrocytes), glutamate accepts free ammonia produced by amino acid catabolism. Glutamine transports ammonia from the peripheral tissues to the liver, where glutamine is broken back to glutamate and ammonia via glutaminase [19, 20]. An increase in free blood ammonia has shown to accumulate glutamine within the astrocytes, which has osmotic effects and causes astrocyte swelling and cerebral edema [21]. The normal rate of urea cycle far exceeds the ammonia production rate by protein catabolism. Therefore, slight elevations in the serum free ammonia concentration (>60 *μ*mol/L) reflect an impairment in the urea cycle, independent of the etiology [19]. This impairment may cause a wide range of neurologic manifestations including asterixis, changes in behavior, slurred speech, sleep disorders, lethargy, stupor, coma, cerebral edema, and even death [18].

Several mechanisms have been proposed in the developing of GaBHA, which seems to be multifactorial [10]. This syndrome occurs almost exclusively in women, and previous reports have found functional ornithine transcarbamylase (OTC) deficiency given a low enzymatic activity [11, 15], even in the presence of normal OTC sequencing genetic testing [11, 15]. Others have reported cases of GaBHA with late-onset carbamoyl phosphate synthase 1 (CPS1) deficiency [15]. Nutritional deficits may lead to functional inhibition of urea cycle enzymes, producing accumulation of ammonia [13]. Low arginine levels inhibit the urea cycle [22] as well as low zinc concentrations, possibly by interfering with the OTC function [23]. Therefore, in adult patients who develop GaBHA, OTC deficiency and other urea cycle abnormalities should be strongly considered. RYGB has been associated with hyperinsulinemia and reactive hypoglycemia, which may contribute to the catabolic state and ammonia excess [24]. Also, the gastric-small bowel pouch in RYGB may cause overgrowth of intestinal flora favoring the production of ammonia by urease-producing bacteria [11].

In general, conservative management of hyperammonemic encephalopathy has been traditionally supportive with prevention of seizures and cerebral edema and lactulose and rifaximin [13], achieving reductions in plasma ammonia levels. Other proposed therapies for GaBHA patients have been the repletion of deficient amino acids, zinc, micro nutrients, and intravenous glucose infusion, attempting to improve the catabolic state [10].

## 5. Conclusion

We present the case of a middle-aged woman without prior liver disease, who developed nonfatal hyperammonemic encephalopathy as a late postoperative complication 20 years after RYGB procedure. GaBHA is an underrecognized potentially fatal syndrome, for which early active treatment may be life-saving. It is thought to be associated with hypoalbuminemia, hypoglycemia, and low zinc plasma levels, indicating severe nutritional deficiency. In high-risk patients, a rigorous screening for plasma ammonia, zinc, and serum albumin may facilitate an early diagnosis and treatment of this entity.

## Figures and Tables

**Table 1:** Laboratory findings during admission.

Laboratory	Day of Admission	Day 3	Day 4 (discharge)	Normal range
Ammonia (*μ*mol/L)	NA	173	24	11 - 50 *μ*mol/L

Hemoglobin (g/dL)	10.3	10.5	10.5	11.1 - 14.6 g/dL

Mean Corpuscular Volume	107.5	106.5	107.9	79.9 - 95.0 fL

Platelets (103/uL)	152	179	341	140 – 400 103/uL

White Blood Cells (103/uL)	7.0	7.5	5.3	4.0 - 10.5 103/uL

Glucose (mg/dl)	81	62	85	65 - 99 mg/dL

Zinc (*μ*g/dL)	54	NA	NA	56-134 *μ*g/dL

Vitamin B1 (nmol/L)	191.9	ND	ND	66.5-200.5 (nmol/L)

Vitamin B12 (pg/mL)	>2,000	ND	ND	232 - 1,245 (pg/mL)

Sodium (mmol/L9	142	145	147	135 - 146 mmol/L

Potassium (mmol/L)	4.1	3.9	3.9	3.5 - 5.5 mmol/L

Chloride (mmol/L)	108	106	108	98 - 110 mmol/L

CO2 (mmol/L)	21	28	25	19 - 34 mmol/L

Anion Gap	13	11	14	6 to 22

Blood Urea Nitrogen (mg/dL)	20	25	30	6 - 20 mg/dL

Osmolality mOsm/Kg	285	291	298	275 - 295 mOsm/kg

Creatinine (mg/dL)	1.09	0.77	0.58	0.40 - 1.10 mg/dL

Calcium, Serum (mg/dL)	7.8	7.8	7.8	8.6 - 10.3 mg/dL

Protein, Total (g/dL)	4.3	4.1	3.9	6.1 - 8.1 g/dL

Albumin (g/dL)	2.6	2.3	1.7	3.5 - 5.2 g/dL

Bilirubin, Total (mg/dL)	0.3	0.5	0.5	0.0 - 1.2 mg/dL

AST (SGOT) (U/L)	66	32	31	10 - 40 U/L

ALT (SGPT) (U/L)	70	55	50	0 - 33 U/L

Alkaline Phosphatase (U/L)	115	122	102	35 - 105 U/L

Magnesium (mg/dL)	1.7	1.6	1.8	1.6 - 2.6 mg/dL

Prothrombin time (sec)	20	NA	NA	12.0 - 14.5 sec

International Normalized Ratio	1.65	NA	1.1	NA

activated partial thromboplastin time (sec)	26.1	NA	NA	23.7 - 35.3 sec

ALT, alanine aminotransferase; AST, aspartate aminotransferase; NA, not available.
